# Supplementary material for: Longitudinal determination of seroprevalence and immune response to SARS-CoV-2 in a population of food and retail workers through decentralized testing and transformation of ELISA datasets
Source: PLoS One. 2024 Dec 16;19(12):e0314499. doi: 10.1371/journal.pone.0314499 (PMC11649073; doi:10.1371/journal.pone.0314499)
Supplement: S1 File — (PDF) [file pone.0314499.s001.pdf]

Supporting information

TITLE: Longitudinal Study on Seroprevalence and Immune Response to SARS-CoV-2 in a Population of Food and Retail Workers Through Transformation of ELISA Datasets

AUTHORS: Abdelhadi Djaïleb[1,2,3 †], Megan-Faye Parker[2,3,4 †], Étienne Lavallée[1,2,3], Matthew Stuible[5], Yves Durocher[2,5], Mathieu Thériault[6,7], Kim Santerre[6,7], Caroline Gilbert[6,7], Denis Boudreau[8,9], Mariana Baz,[6,7] Jean-Francois Masson[1,10,11], Marc-André Langlois[12,13], Sylvie Trottier[6,7], Daniela Quaglia[1,2,3,4,14] and Joelle N. Pelletier[1,2,3,4]*

[1]Département de Chimie, Université de Montréal, Montréal, QC, Canada ; [2]PROTEO, Regroupement Québécois de Recherche sur la Fonction, l'Ingénierie et les Applications des Protéines, Québec, QC, Canada ; [3]Centre en Chimie Verte et Catalyse, Université de Montréal, Montréal, QC, Canada; [4]Départment de Biochimie et Médecine Moléculaire, Université de Montréal, Montréal, QC, Canada; [5]Mammalian Cell Expression, Human Health Therapeutics Research Centre, National Research Council Canada, Montréal, QC, Canada; [6]Centre de Recherche du Centre Hospitalier Universitaire de Québec, Université Laval, Québec, QC, Canada; [7]Département de Microbiologie-Infectiologie et d'Immunologie, Université Laval, Québec, QC, Canada; [8]Départment de Chimie, Université Laval, Québec, QC, Canada; [9]Centre d'Optique, Photonique et Laser, Université Laval, Québec, QC, Canada; [10]Institut Courtois, Université de Montréal, QC, Canada; [1111]Centre Québécois sur les Matériaux Fonctionnels, Regroupement québécois sur les matériaux de pointe, Centre Interdisciplinaire de Recherche sur le Cerveau et l'Apprentissage, Montréal, QC, Canada; [12]Department of Biochemistry, Microbiology and Immunology, Faculty of Medicine, University of Ottawa, Ottawa, ON, Canada; [13]Ottawa Center for Infection, Immunity and Inflammation (CI3), Ottawa, ON, Canada; [14] Département de Chimie, Université du Québec à Montréal, Montréal, QC, Canada

† These authors contributed equally

* Corresponding author information: joelle.pelletier@umontreal.ca

Table of contents

Supporting Tables

S1 Table. Number of datapoints removed for each antigen dataset.

S2 Table. Percentage of fully vaccinated (at least two doses) individuals per visit.

S3 Table. Sensitivity and specificity of the ELISA assays.

Supporting Figures

S1 Figure. Seropositivity determined with in-house ELISA over 18 months.

S2 Figure. Seropositivity determined with centralized ELISA over 18 months.

S3 Figure. Overview of the ELISA IgM results over 18 months.

S4 Figure. Comparative assessment of humoral immune response in unvaccinated and vaccinated individuals.

S5 Figure. Comparative assessment of humoral immune response for different vaccine types.

S6 Figure. Impact of public transport use on immune response.

S7 Figure. Impact of being in contact with a COVID-19 positive person on immune response.

S8 Figure. Impact of BMI on immune response.

S1 Table. Number of datapoints removed for each antigen dataset.

	Ancestral spike		Nucleocapsid		Delta spike		Omicron spike	
Visit	Data points removed	Total data points	Data points removed	Total data points	Data points removed	Total data points	Data points removed	Total data points
V1	1	303	12	303	56	304	6	304
V2	7	297	34	294	19	297	36	297
V3	2	291	12	292	3	289	1	290
V4	0	194	2	192	-	-	0	194
V5	1	191	2	182	-	-	0	194

S2 Table. Percentage of fully vaccinated (at least two doses) individuals per visit.

Visit	Participants having received at least 2 vaccine doses	Participants having completed the visit	Fully vaccinated participants (%)
V1	146	304	48
V2	182	297	61
V3	188	291	65
V4	188	198	95
V5	188	194	97

S3 Table. Sensitivity and specificity of the ELISA assays.

Model		Reference ELISA		
		Positive	Negative	Total
In-House ELISA	Positive	TP	FP	TP+FP
	Negative	FN	TN	FN+TN
	Total	TP+FN	FP+TN	

TP = True Positive [a]

FP = False Positive [b]

FN = False Negative

TN = True Negative

[a] True Positive: Samples found to be positive according to the centralized serological assay combined with a positive PCR or antigenic test within 60 days preceding the serological assay.

[b] True Negative: Samples found to be negative according to the centralized serological assay combined with negative PCR or antigenic test.

Sensitivity and specificity of in-house, decentralized ELISA:

Vaccine Immunity		Reference ELISA		
		Positive	Negative	Total
In-House ELISA	Positive	1027	54	1081
	Negative	136	58	194
	Total	1163	112	

Sensitivity = TP / (TP + FN) = 0.88

Specificity = TN / (FP + TN) = 0.52

Natural Immunity		Reference ELISA + Positive COVID TEST within 60 days + Participant feedback		
		Positive	Negative	Total
In-House ELISA	Positive	210	187	397
	Negative	98	780	878
	Total	308	967	

Sensitivity = TP / (TP + FN) = 0.68

Specificity = TN / (FP + TN) = 0.81

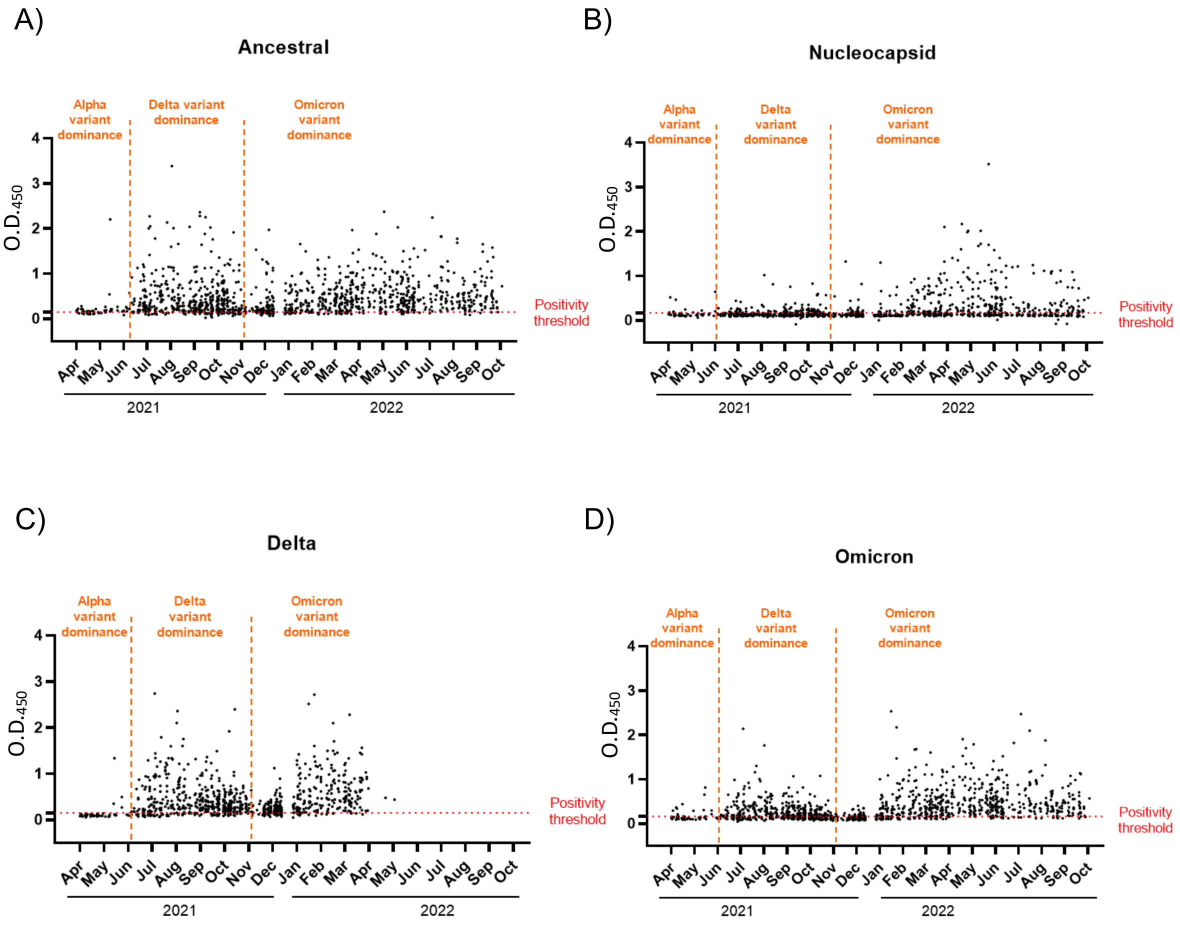

S1 Figure. Seropositivity determined with in-house ELISA over 18 months. Determination of IgG for the **A)** ancestral spike, **B)** nucleocapsid, **C)** delta spike and **D)** Omicron antigens were measured. On the x-axis: the month of reference. On the y-axis: $O.D._{450}$ for each sample measured.

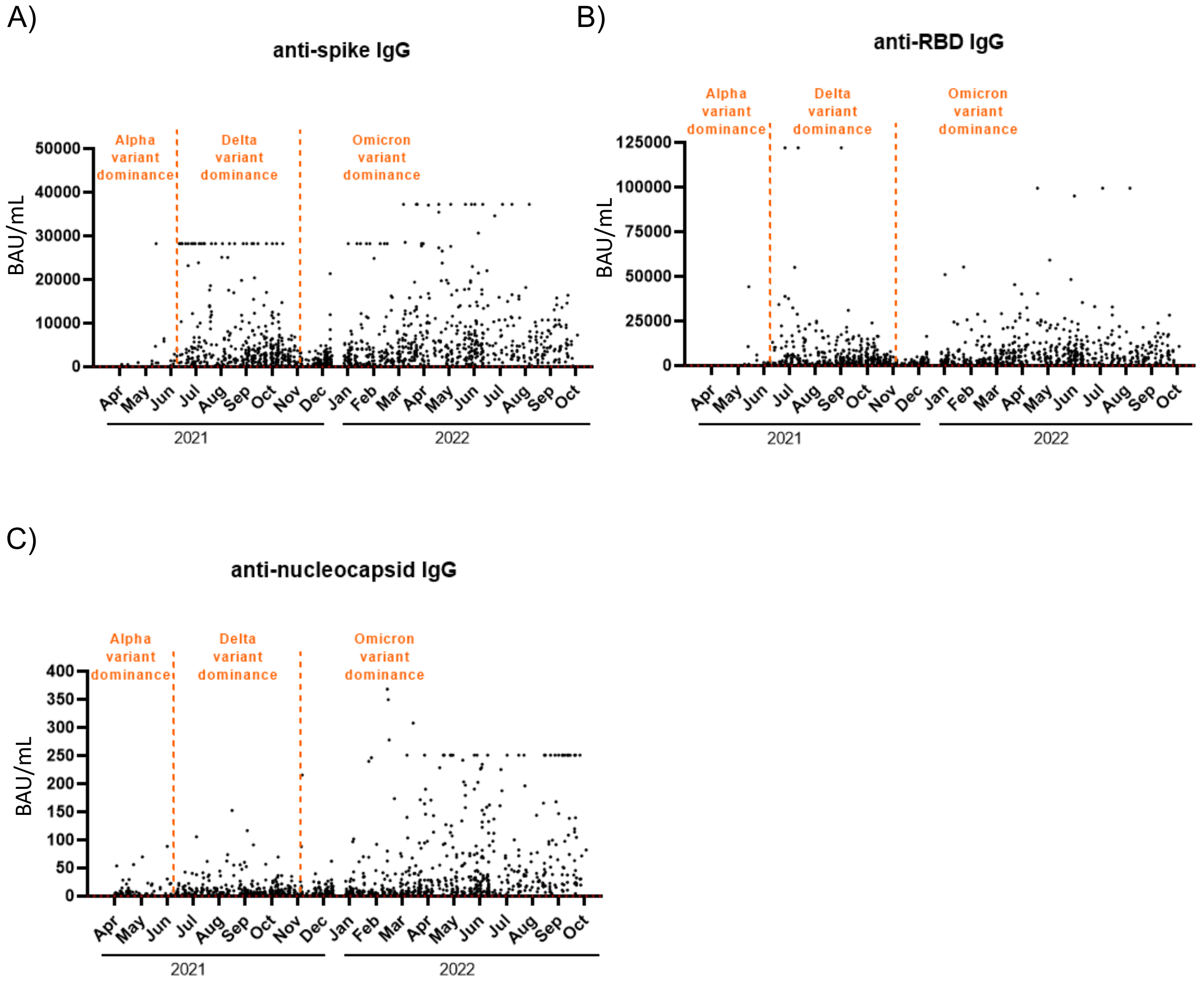

S2 Figure. Seropositivity determined with centralized ELISA over 18 months. Determination of IgG for the **A)** ancestral spike, **B)** receptor binding domain (RBD) and **C)** nucleocapsid antigens were measured. On the x-axis: the month of reference. On the y-axis: the concentration in BAU/mL for each sample measured.

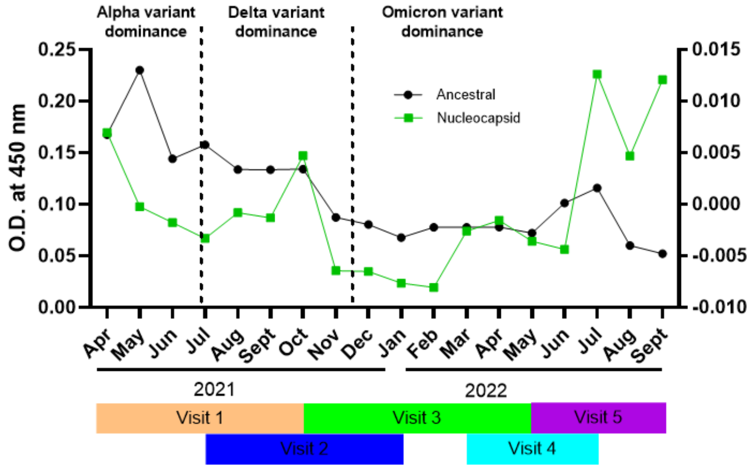

S3 Figure. Overview of the ELISA IgM results over 18 months. Sample collection visits are shown in different colors under the graph. IgMs for the ancestral spike ectodomain and the nucleocapsid antigen were measured (see legend). On the x-axis: the month of reference. On the y-axes: the mean O.D.$_{450}$ of all samples collected during each given month. Note the different scale on the y-axes.

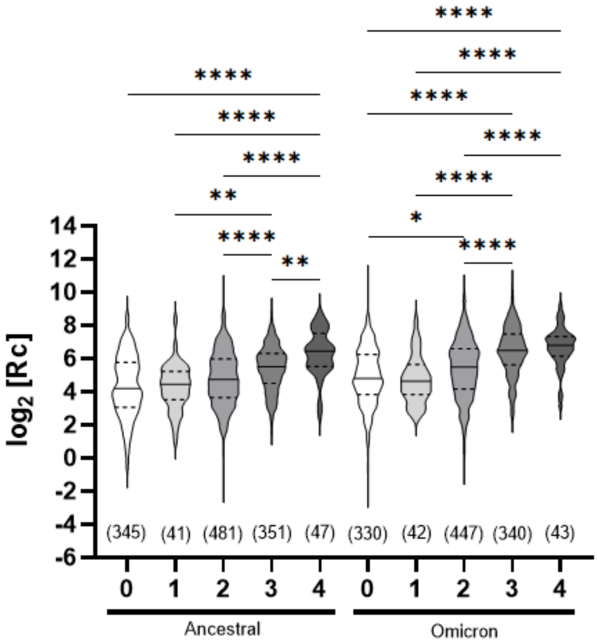

S4 Figure. Comparative assessment of humoral immune response in unvaccinated and vaccinated individuals including that of individuals following seroconversion for anti-nucleocapsid IgG. ELISA assays measuring ancestral or Omicron anti-spike IgG levels were analyzed collectively. X-axis: number of vaccine doses received (minimum 7 days post-vaccination). Each grouping includes all data collected (n is given) for that number of vaccine doses, including any data points taken at different visits for the same individual whose vaccination status did not change. For this reason, the number of datapoints may exceed the total number of participants (n = 304). y-axis: data points after logarithmic transformation. The median (solid line) and quartiles (dashed lines) are shown in the violin plots; no outliers were removed. Statistical significance: *, $p < 0.05$; **, $p < 0.01$; ****, $p < 0.0001$.

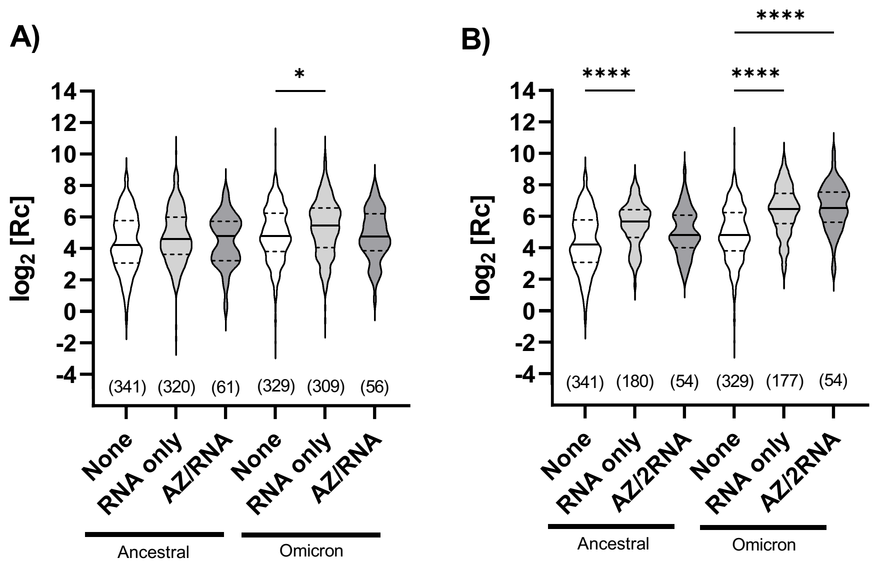

S5 Figure. Comparative assessment of humoral immune response for different vaccine types including that of individuals following seroconversion for anti-nucleocapsid IgG. ELISA assays measuring ancestral or Omicron anti-spike IgG levels were analyzed collectively. Samples following administration of **A)** 2 vaccine doses or **B)** 3 vaccine doses. “RNA only”: either the Pfizer-BioNTech Comirnaty® mRNA COVID-19 vaccine, the Moderna Spikevax® mRNA COVID-19 vaccine, or a combination of both. “AZ/RNA” and “AZ/2RNA”: one dose of the AstraZeneca COVISHIELD® viral vector-based COVID-19 vaccine and one or two doses of an RNA vaccine, respectively. Each grouping includes all data collected (n is given) for that type of vaccine, including any datapoints taken at different visits for the same individual whose vaccination status did not change. For this reason, the number of datapoints may exceed the total number of participants (n = 304). Individuals who had received the AstraZeneca vaccine only, or two doses of AstraZeneca and one of an RNA vaccine, were excluded as there was insufficient data to allow statistical analysis. y-axes: data points after logarithmic transformation. The median (solid line) and quartiles (dashed lines) are shown in the violin plots; no outliers were removed. Statistical significance: *, $p < 0.05$; ****, $p < 0.0001$.

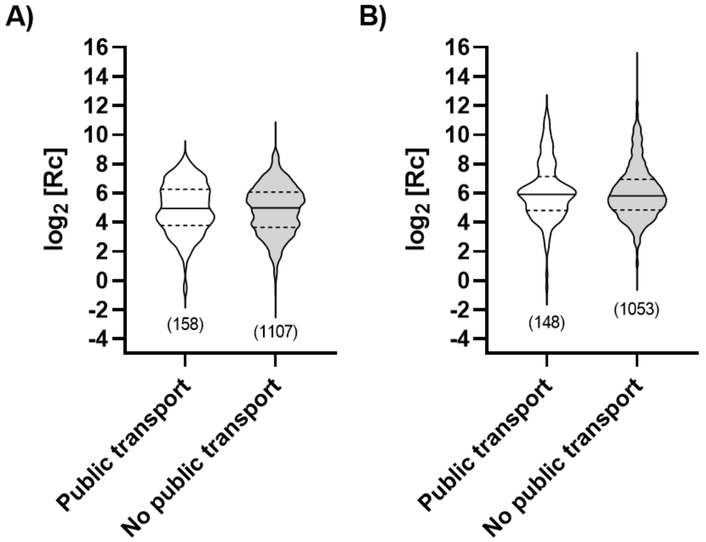

S6 Figure. Impact of public transport use on immune response. ELISA measuring IgG for the **A)** ancestral variant spike ectodomain and **B)** nucleocapsid protein performed from visits 1 through 5 are analyzed together. Results are shown in post-transformation logarithmic form. The median (solid line) and quartiles (dashed lines) are shown in the violin plot where all values are included (no outliers removed). The number of data points (n) is shown in brackets below each dataset.

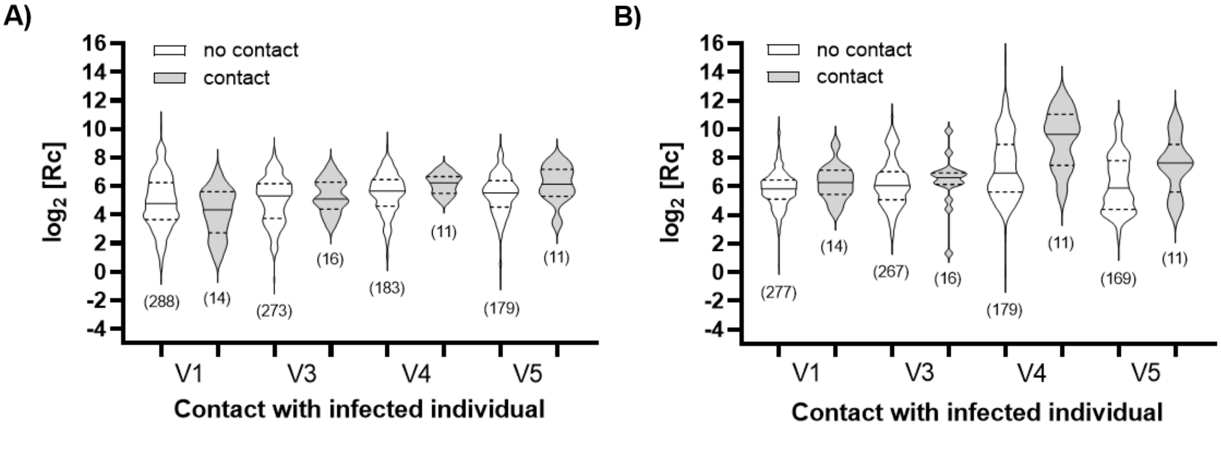

S7 Figure. Impact of being in contact with a COVID-19 positive person on immune response. ELISA measuring IgG for the **A)** ancestral variant spike ectodomain and **B)** nucleocapsid protein performed from visits 1 through 5; visit 2 is not shown as there are not enough datapoints for statistical analysis. Results are shown in post-transformation logarithmic form. The median (solid line) and quartiles (dashed lines) are shown in the violin plot. The number of data points (n) is shown in brackets below each dataset.

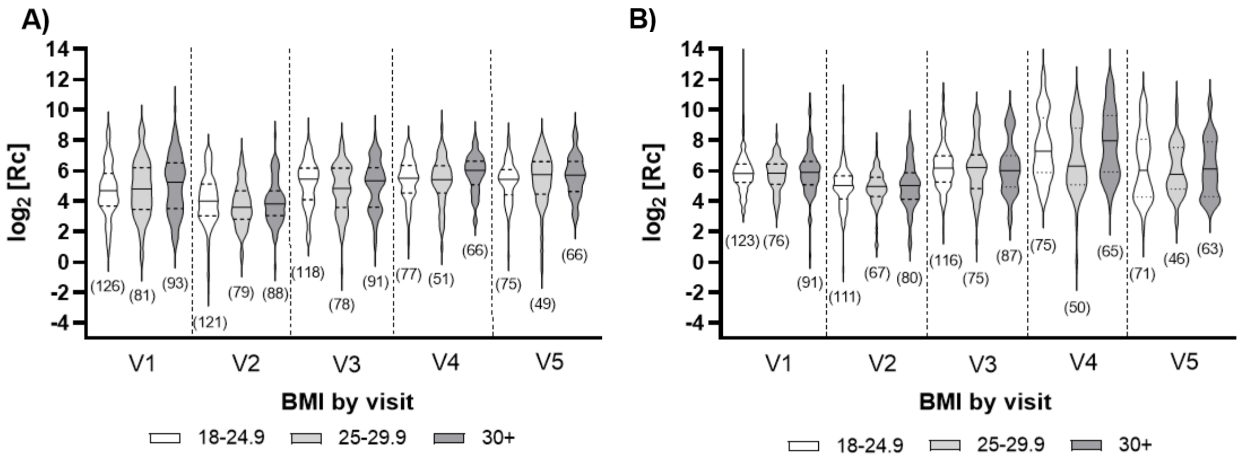

S8 Figure. Impact of BMI on immune response. ELISA measuring IgG for the **A)** ancestral variant spike ectodomain and **B)** nucleocapsid protein performed from visits 1 through 5. Results are shown in post-transformation logarithmic form. The median (solid line) and quartiles (dashed lines) are shown in the violin plot. The number of data points (n) is shown in brackets below each dataset. Two datapoints (BMI < 18) were removed as the category did not have enough datapoints to allow statistical analysis.
